# Contactless and spatially structured cooling by directing thermal radiation

**DOI:** 10.1038/s41598-021-95606-2

**Published:** 2021-08-10

**Authors:** Nicola M. Kerschbaumer, Stefan Niedermaier, Theobald Lohmüller, Jochen Feldmann

**Affiliations:** Chair for Photonics and Optoelectronics, Department of Physics, Nano-Institute Munich, Ludwig-Maximilians-Universität (LMU), Königinstraße 10, 80539 Munich, Germany

**Keywords:** Applied optics, Optical techniques, Thermodynamics

## Abstract

In recent years, radiative cooling has become a topic of considerable interest for applications in the context of thermal building management and energy saving. The idea to direct thermal radiation in a controlled way to achieve contactless sample cooling for laboratory applications, however, is scarcely explored. Here, we present an approach to obtain spatially structured radiative cooling. By using an elliptical mirror, we are able to enhance the view factor of radiative heat transfer between a room temperature substrate and a cold temperature landscape by a factor of 92. A temperature pattern and confined thermal gradients with a slope of ~ 0.2 °C/mm are created. The experimental applicability of this spatially structured cooling approach is demonstrated by contactless supercooling of hexadecane in a home-built microfluidic sample. This novel concept for structured cooling yields numerous applications in science and engineering as it provides a means of controlled temperature manipulation with minimal physical disturbance.

## Introduction

Temperature is a fundamental regulating factor for most biological, chemical, and physical processes. Many properties of living cells, for example, such as stiffness^1^, gene expression levels^2^, membrane permeability^3^, or cell activation thresholds^4^ are influenced by local temperature gradients. Moreover, the rates of chemical reactions, phase transitions and the physical properties of materials such as conduction or magnetic behavior are influenced by temperature.

Light is ideally suited for temperature manipulation given its inherent properties of being contactless and easy to manipulate. Notably, radiative temperature manipulation is predominantly discussed in the context of heating. Photothermal effects, i.e. the generation of heat via photoexcitation, have been extensively studied and applied to generate temperature gradients^5,6^, to control convection^7–9^ or thermophoresis^10–13^ and to enhance the efficiency of chemical^14,15^ and catalytic reactions^16,17^. In this regard, plasmonic heating^18,19^ is a well-known example, where laser heating of individual gold^20,21^ or silver^22^ nanoparticles has led to important technological advancements, such as photothermal therapy^23,24^, fast DNA melting and PCR^25,26^ or water purification^27^.

However, the idea to cool objects via radiative heat transfer by emission has moved more in the scientific focus in recent years. For example, Shanhui Fan and other groups have studied how radiative cooling enables consumption-less temperature control of houses^28–34^. By designing nanophotonic substrates that emit thermal radiation in the range of the atmospheric infrared window (between ∼8 and ∼13 μm), where the absorption by atmospheric gases is low, they achieved substrate cooling via thermal energy exchange with the cold universe, even in broad daylight.

Inspired by these research works, we considered whether radiative energy transfer can be applied to obtain spatially structured cooling and temperature gradients of relevant temperature changes exceeding 5 °C in a similar way as it has been shown for heating. In fact, radiative cooling of nanostructures in the near-field has been shown theoretically^35,36^ and experimentally^37^. However, in these cases the samples had to be aligned at a close distance, which was much smaller than the thermal radiation wavelength. The possibility to achieve contactless and spatially structured radiative cooling also in the far-field has not been demonstrated to date. The challenge to achieve such a type of cooling relies on developing an efficient strategy to collect all the thermal radiation emitted from a warm sample and to direct it to a well-defined low-temperature landscape.

As a short reminder, the amount of thermal radiation emitted by a body is dependent on its temperature, according to Planck’s law^38^:

1 $$\begin{array}{*{20}c} {I_{BB} \left( {T, \lambda } \right) = \frac{{2\pi hc^{2} }}{{\lambda^{5} }}\frac{1}{{e^{{\frac{hc}{{\lambda kT}}}} - 1}}} \\ \end{array}$$

When integrating this intensity over all wavelengths and over the solid angle of a half-sphere, the emitted power is given in form of the Stefan–Boltzmann law^39^:

2 $$\begin{array}{*{20}c} {P = \varepsilon A\sigma T^{4} } \\ \end{array}$$

**Figure 1:**
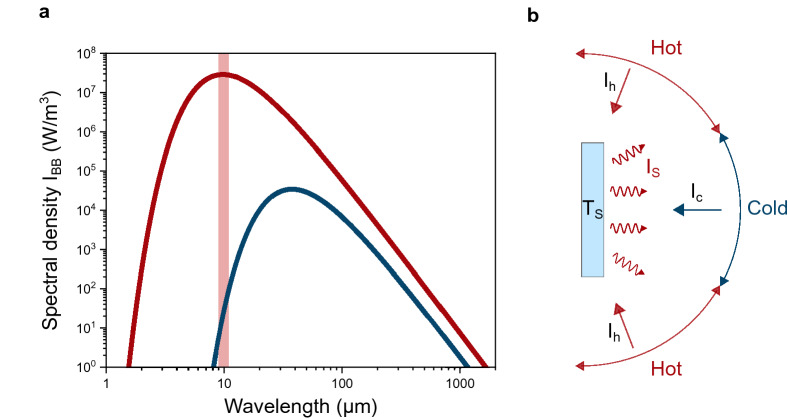
Black body emission and view factor. (**a**) Blackbody radiation spectra at room temperature (red curve) and at liquid nitrogen temperature (blue curve). The light red bar indicates the wavelength range relevant for this work (9–11 µm). (**b**) While a sample will always emit thermal energy homogeneously within the solid angle of a half-sphere, the amount of incident hot and cold radiation will determine the temperature distribution on the sample. By tuning the view factor of hot and cold radiation over the solid angle, the temperature profile can be manipulated. The sample is at a temperature T_S_ and emits radiation with intensity I_S_. The hot sections of the solid angle environment emit with intensity I_h_ and the cold section with I_C_.

Wien's displacement law^40^ further states that the radiation of a blackbody has a wavelength maximum that depends on its temperature. The blackbody radiation curves for 295 K and for 77 K (boiling point of liquid nitrogen) are shown in Fig. 1a. For T = 295 K the peak is at λ_max_ = 9.8 µm. For T = 77 K, the peak is red-shifted to λ_max_ = 37.6 µm, but more importantly, the intensity of thermal radiation of the body at room temperature is a factor 10^5^ higher. This means the direction of energy dissipation from hot to cold is dominating and causing the hot body to decrease in temperature.

Besides the temperature difference, the efficiency of radiative heat transfer between two physical objects is highly dependent on the so-called shape or view factor^41,42^. The view factor is a purely geometrical measure that describes the emission angle of thermal radiation from a warm surface that will be intercepted by a cold object. As schematically shown in Fig. 1b, this may only be a fraction of the emitted radiation. Depending on the sample geometry, the substrate will exchange thermal energy also with other segments within the solid angle that have the same temperature or could be even hotter.

Taken together, these considerations already illustrate that efficient directing and focusing of thermal radiation from a hot to a cold body cannot be achieved with a simple lens. In that case, the limited numerical aperture results in a small angle over which thermal radiation is collected. Furthermore, re-radiation from the environment covered by the solid angle will limit the overall cooling efficiency. The realization of structured radiative cooling therefore requires an optical system that collects the thermal radiation from almost the entire solid angle and directs it onto a cold region.

Here, we report an experimental strategy to achieve small-scale thermal pattern generation by manipulating the view factor of thermal radiation for spatially confined radiative cooling. In our setup, we position one sample at room temperature in the first focal point of an elliptical mirror. The second focal point is placed onto a highly absorbent sample that is mounted inside a liquid nitrogen cooled cryostat. Without the mirror, the view factor in this configuration merely consists of a room temperature environment with a cold central spot, which we refer to as “cold bull’s eye”. The elliptical mirror, however, provides a > 90 times enhancement of the view factor and enables spatially structured cooling of the room temperature sample. We exemplify the applicability of our approach by demonstrating a proof-of-principle experiment for contactless radiative supercooling of hexadecane in a microfluidic chip.

## Results

### Structured radiative cooling

**Figure 2:**
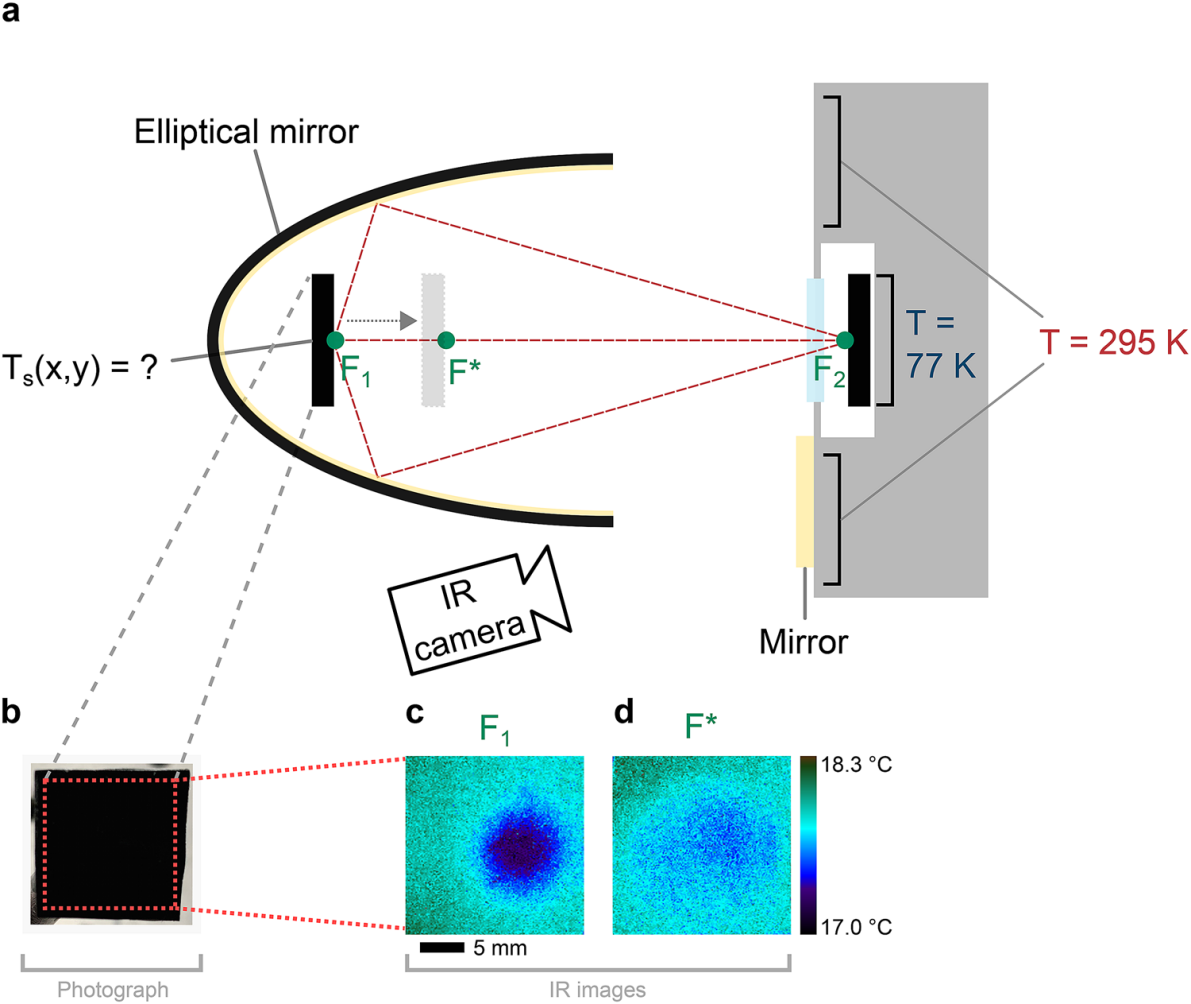
Schematic of the setup and resulting cooling pattern. (**a**) An elliptical mirror collects the thermal radiation emitted by a room temperature sample positioned at the focus point F_1_. The radiation is focused onto a sample at the second focal point F_2_, which is placed inside the cold region of a cryostat. A germanium window transparent for IR radiation (9–11 µm) seals the cryostat (SI, Figure S1). An additional mirror allows simultaneous temperature measurements with an IR camera. (**b**) Photograph of the “metal velvet” sample. (**c**) IR camera image of the sample at position F_1_. (**d**) IR camera image of the sample at position F*. The localized cold area vanishes when the substrate is placed out of focus.

A schematic of the setup is shown in Fig. 2a. A sample is initially at room temperature (T_S,i_ = 295 K) and positioned in the first focal point F_1_ of the elliptical mirror. The second focus F_2_ at 101.6 mm away, is placed onto a temperature landscape consisting of the cold bull’s eye of the cryostat sample (T ≈ 77 K) embedded in an ambient environment. According to the Stefan–Boltzmann law^39^, the total radiation power of the room temperature sample is P_w_ = 169 mW. The power radiated from the cold central region is only P_c_ = 785 µW. As mentioned above, these values correspond to the integrated area of the spectral densities plotted in Fig. 1a, where the red bar indicates the mid-IR wavelength range from 9 to 11 µm, which corresponds to the temperature range relevant for our experiments. In this window the sample and the room temperature section of the view factor have an emission intensity which exceeds the intensity of the cold bull’s eye by a factor of 10^5^.This large difference drives the net transfer of thermal energy from the room temperature sample to the cold bull’s eye.

An IR thermal camera is used for temperature measurements and to analyze temperature distributions on the sample (Fig. 2b) during the experiment. Furthermore, several thermocouples are positioned within 30 cm around the setup as well as in between the elliptical mirror and the cryostat. These thermocouples are used to constantly monitor the room temperature and to ensure that convection does not contribute significantly to the cooling process. In the following, the temperature of the cryostat will be given in Kelvin, while the sample temperatures will be discussed in Celsius. This designation is chosen to enhance clarity of the process description.

At the beginning of the experiment, the optical path between the sample and the structured temperature landscape is blocked with a metal shield. In this situation, no temperature change is observed, since the sample is in a thermal equilibrium with the environment. When the shield is removed, however, a cold spot appears in the thermal camera image within seconds (Fig. 2c). The sample temperature is now dependent on the lateral position on the sample, i.e. a temperature profile T_S_(x,y) is obtained. This cooling profile is the result of radiative heat transfer since the thermal radiation emitted in all directions at the point F_1_ is now focused on the cold central region behind the cryostat window. Notably, the cold spot vanishes when the sample at F_1_ is moved to a different position F* along the major axis of the elliptical mirror (Fig. 2d). This control experiment confirms the concept of spatially structured radiative cooling and illustrates how the elliptical mirror shapes the view factor between both samples. At position F* only a small fraction of the thermal radiation is directed onto the cryostat window. Therefore, the cold spot smears out. At F* (or without an elliptical mirror) both samples exchange thermal energy between their opposing surface areas, since they are aligned face-to-face. This component of radiative cooling is always constant. The elliptical mirror, however, improves the view factor between point F_1_ and the cold bull’s eye significantly by collecting the thermal radiation from over 72% of the solid angle. For the setup geometry and conditions shown in Fig. 2a, this corresponds to a 92-fold increase of the area of the view factor (see also Supporting Information). This is a major enhancement by almost 2 orders of magnitude.

At this point, one must keep in mind that radiative heat transfer is not the only process that contributes to the temperature distribution shown in Fig. 2c. Conduction and convection occur simultaneously and are competing against the radiative heat transfer. Radiative cooling is strongest in the focus point of the elliptical mirror, which is in the center of the sample. Heat diffusion from the sample edges (i.e., conduction) and contact to the surrounding air at room temperature (i.e., convection), however, hamper and limit the cooling efficiency.

### Temperature gradient and cooling efficiency

To analyze the influence of conduction, we repeat the previous experiment while changing the cryostat temperature. Again, the sample is placed at the focal point F_1_, while the second focal point F_2_ is positioned at the cold bull’s eye. This time, a glass substrate coated with black paint is used as an emitter sample to better visualize temperature patterns, since glass is thermally less conductive than the metal oxide coated aluminum foil.

**Figure 3:**
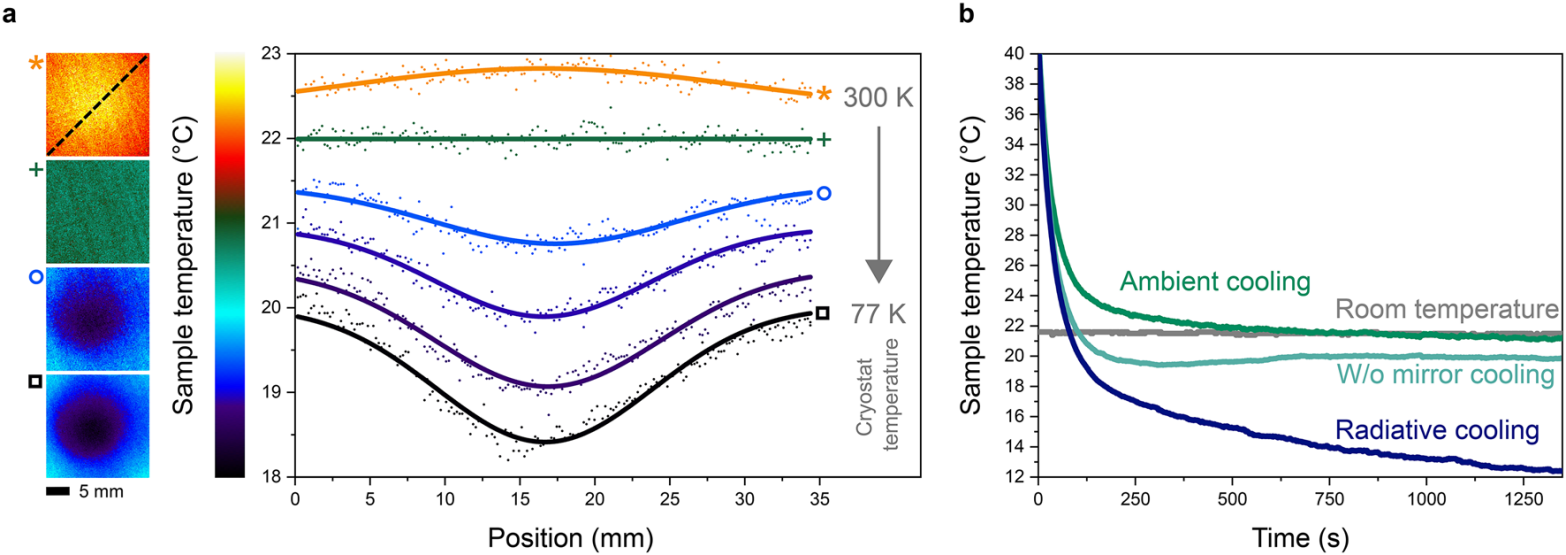
Temperature landscape variation and cooling efficiency. (**a**) Four IR images of the sample show the temperature distribution across the sample surface at four exemplary points in time during the radiative cooling experiment. The temperature profile has been extracted from the sample diagonal for various IR images (indicated by dashed line in the IR image on the top) and the respective distributions are plotted in the graph. Symbols correlate the curves to the images. During the cooling experiment, the central cryostat temperature was decreased from 300 to 77 K. (**b**) Comparison of the cooling process in the center of the sample for ambient conditions (green curve) with a cooling rate of 17.5 mK/s, cooling with the cryostat but without the mirror (petrol curve) radiative cooling (blue curve) with a cooling rate of 26.4 mK/s. Throughout the measurement, the room temperature was monitored as a reference (grey curve).

The sample inside the cryostat is initially heated up to 300 K and subsequently cooled down to 77 K over the course of 45 min. The evolution of the substrate’s temperature distribution T_s_(x,y) for decreasing temperatures of the bull’s eye region is shown in Fig. 3a. For 300 K, the temperature profile of the sample at F_1_ shows a small peak at the focal point. This indicates that localized radiative heating rather than cooling is taking place because the view factor now contains a central hot region with a temperature higher than the sample temperature. Heat generated in the focus now flows to the edges, where the temperature is also slightly elevated. It further illustrates, that the direction of radiative heat transfer can be reversed in our setup.

When the cryostat is cooled down to room temperature, no thermal gradient is observed since the sample and the temperature landscape on the other side of the focusing system are in thermal equilibrium. For decreasing cryostat temperatures, the temperature profile starts to show a dip, which is becoming more pronounced the further the temperature goes down. Furthermore, one observes that the temperature at the sample edges is also decreasing over time. This indicates that during the cool down of the bull’s eye, the whole sample at F_1_ loses thermal energy via the radiative channel, since heat flows constantly from the sample edges to the focus point. This heat flow also determines the size of the cold spot which has a FWHM of roughly 11 mm (in Fig. 2c) and thus the focusing capabilities of the system. Elliptical mirrors themselves may also suffer from spherical or chromatic aberrations. However, since we are not tightly focusing the radiation and are working with large focal spots, aberrations can be neglected.

To discriminate between the efficiency of radiative cooling and the influence of conduction or convection, we compared the cooling rates of our sample with and without a structured temperature landscape. Conduction and convection are both accurately described by Newton’s law of cooling^43^. It states that the temperature decay of a warm object that cools down to the temperature of the environment is exponential over time. Energy transfer by radiation, however, is best described by the Stefan–Boltzmann law^39^ as mentioned earlier. A cooling process driven by radiative heat transfer should therefore deviate from Newton’s law and be accelerated for a higher temperature difference.

We heated the metal oxide sample to 40 °C and placed it at the point F_1_ in the ellipse. At first, we analyzed the ambient cooling of the sample when all sections of the temperature landscape are at room temperature. The measured temperature decrease over time, which corresponds to a cooling rate of *κ*_*ac*_ = 17.5 mK/s, is shown in Fig. 3b. The mono-exponential fit of the temperature curve shows a high R^2^ value (coefficient of determination) of 0.97, which indicates a nearly perfect mono-exponential association between heat-dissipation and time. The ambient cooling process thus obeys Newton’s law of cooling, where conduction and convection are the dominating pathways.

The same experiment is now repeated with the cold bull’s eye cooled down to 77 K. Again, the temperature decrease over time is measured at the center of the sample. We discover a higher cooling rate of *κ*_*rc*_ = 26.4 mK/s when the thermal radiation of the sample is focused onto the central cold region (Fig. 3b). The corresponding R^2^ value for the mono-exponential fit of the radiative cooling trace is only 0.91 and the temperature decay therefore deviates from Newton’s cooling law. This shows the significant contribution of radiative heat transfer to the overall cooling process and that the cooling is accelerated by the radiative component.

Notably, the sample cooled down radiatively and reached a final temperature of approximately 12 °C. At this point, conduction from the sample mounts or natural air convection apparently prevents any further cooling through the radiative channel and all three processes are in equilibrium. However, the final temperature that is reachable by radiative cooling will strongly depend on the specific setup and the overall experimental conditions. Especially the temperature landscape presented to the sample defines the cooling efficiency and thermal pattern. This can be exemplified by a second control experiment, where we now place a pre-heated sample in front of the cold cryostat but remove the elliptical mirror. In this case, the two samples facing each other can still exchange thermal energy but the view factor is solely determined by the small solid angle between the two surface areas. This pre-heated sample homogeneously cooled down just below room temperature to only ~ 20 °C. The cooling in this case stems partly from the radiative component and residual convection effects.

One must also keep in mind, that the emission wavelength and the cooling power are both temperature dependent according to the Wien and Stefan–Boltzmann laws^39^. The cooling power in particular has a T^4^ dependence (Eq. 2). The cooling power is therefore also reduced as the sample cools down. A temperature difference of ~ 30 °C, as shown in our experiment, already corresponds to a cooling power difference of 71 mW.

### Radiative supercooling of hexadecane

So far, we have established that directed radiative cooling works contactless and does not require specific vacuum conditions. This emphasizes that such radiative cooling may be well suited for implementation in laboratory experiments, where cooling via standard approaches such as thermoelectric devices, liquid or gaseous coolants or ventilation is challenging or maybe even impossible. For example, the controlled cooling of liquids below their freezing point, so called super- or undercooling, demands highly quiescent experimental conditions^44^, since any slight mechanical or electrical disturbance of the system can initiate spontaneous nucleation and sample freezing. Supercooling occurs naturally in plants^45^ and animals^46^ that inhabit regions of extreme temperatures but has been discussed for applications in medicine^47–49^, drug delivery^44^, food preservation^50^, and energy storage^51^. The experimental realization of supercooling, however, is often challenging since supercooled liquids are notoriously unstable^52^.

Heat transfer by thermal radiation is ideally suited for supercooling. No physical contact with a sample is required, which provides minimal disturbance. To demonstrate this idea, we looked into the possibility of radiative supercooling of hexadecane.

**Figure 4:**
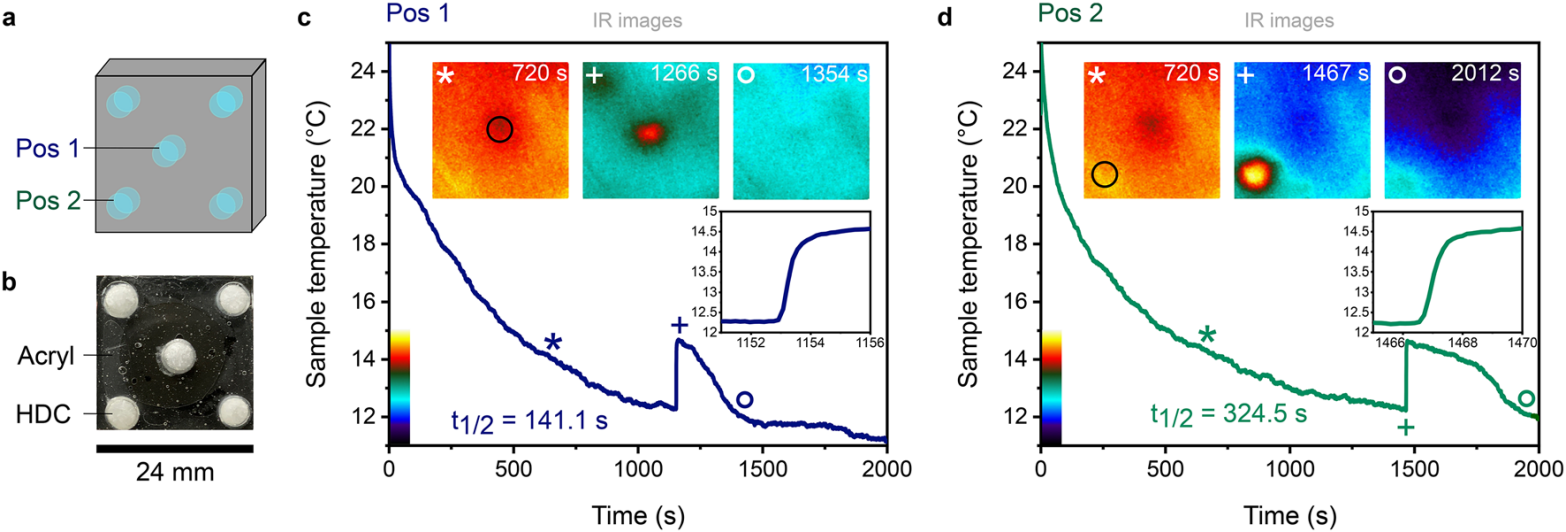
Supercooling of hexadecane. (**a**) Schematic of the home-built microfluidic chip. (**b**) Photograph of the sample with hexadecane (HDC) in the solid phase in the five chambers. (**c**) Cooling curve of the central chamber (Pos 1). After 200 s the melting temperature of hexadecane at 18 °C is reached. A sharp increase in temperature is observed after 1150 s, which indicates a liquid–solid phase transition of hexadecane. The phase transition peak has a half-lifetime of 141.1 s until the sample has reached the same temperature as before freezing. Inset: Zoom-in showing a temperature increase from 12.3 to 14.6 °C between 1152 and 1154 s. IR images (scale bar as in **b**) show the temperature distribution on the sample surface for different time frames. The black circle in the first image indicates the area used to extract the temperature values over time (blue curve). (**d**) Cooling curve measured for Pos 2. The half-lifetime of the phase transition peak of t_1/2_ = 324.5 s is longer compared to t_1/2_ for Pos 1. Inset: Zoom-in showing the temperature increase from 12.3 to 14.6 °C between 1466 and 1468 s.

For this experiment, we designed a microfluidic chip with five chambers arranged like the five-point face of a dice (Fig. 4a, b). Each chamber can be completely filled with 30 μL hexadecane and the sample is sealed from both sides with a glass cover slide. To conduct the measurement, the chip is mounted in such a way, that the focal point of the elliptical mirror is located only at the central chamber, while the other 4 chambers are not in focus. The cooling rate at the central spot (Pos 1) is analyzed by temperature measurements with the IR camera (Fig. 4c). After ~ 3 min of cooling, the temperature of the central chamber reached the melting point of hexadecane. However, no phase transition was observed and the hexadecane remained in a liquid phase as the cooling progressed further. When the temperature reached 12.2 °C, a sudden temperature increase to ~ 15 °C was observed, indicative for spontaneous crystallization. The solidification of hexadecane is also visible by the naked eye since the hexadecane inside the chamber, which is transparent when liquid, immediately turns opaque (Fig. 4b). After the phase transition, the temperature of the solid hexadecane decreased again due to the ongoing radiative cooling, until a final temperature of ~ 11 °C was reached. The half-lifetime for the sample to reach the temperature again at which supercooling occurred is t_1/2_ = 141.1 s (details are provided in the Supporting Information).

The jumps in the sample temperature observed in our measurement are in good agreement with previous reports on supercooling of hexadecane. In general, supercooling of liquids can only be achieved down to a temperature were homogeneous nucleation occurs and spontaneous crystallization takes place. For hexadecane droplets, Abramov et al.^53^*.* reported an onset temperature for crystallization of 12.9 ± 0.5 °C, about 6 °C below the melting point, which is in very good agreement with our data. Crystallization is an exothermic process because molecules have more kinetic energy in a liquid than in a solid. The phase transition from liquid to solid is therefore characterized by a sharp temperature increase. The onset temperature for the liquid–solid phase transition was reported to be at 15.66 ± 0.32 °C, about three degrees below the melting point. Again, this is in very good agreement with our temperature recordings and confirms the controlled hexadecane supercooling by radiative energy transfer.

Notably, supercooling and the corresponding liquid–solid phase transitions are also observed in reservoir chambers outside of the focus, after 22 min (Fig. 4d, Pos 2). As mentioned before, the cooling of the sample edges is observed in all of our experiments, due to thermal dissipation within the entire sample. Like in the experiment shown in Fig. 3a, heat flows from the sample edges to the center, which eventually cools down the outer reservoirs, as well. However, the cooling rate for liquid hexadecane in the central chamber (*κ*_1_ = 138.0 mK/s) is five times faster than the cooling rate at Pos 2 (*κ*_1_ = 24.1 mK/s). Also, in the solid phase, the chamber at Pos 2 cools with a half-lifetime of t_1/2_ = 324.5 s back to the phase transition temperature, which is twice as long as the half-lifetime observed for the chamber in Pos 1. Cooling then continued, until a final temperature of ~ 12 °C was reached at Pos 2 and the sample was at equilibrium. This shows the strong influence of directed radiative cooling, even for solid hexadecane, where thermal conductivity is expected to be stronger than in the liquid.

For more information about the applied fits and the peaks, refer to Figure S3, Supporting Information. The overall cooling dynamics are a competition between radiative heat transfer on the one hand and conduction as well as convection on the other hand. In our supercooling experiment, the heat transfer between the central and the outside chambers could be further controlled by choosing a different sample design. Thermally isolating the fluid chambers in low heat conducting materials or separating them with pockets of air, for example, could significantly alter the heat diffusion within the substrate.

## Discussion

In summary, we have shown that spatially structured cooling of a sample with a lateral temperature profile is efficiently achieved by manipulating the view factor of thermal radiation. We use an elliptical mirror with two foci to obtain thermal energy transfer between a room temperature sample and a cold bull’s eye within a room temperature environment. Although competitive conduction and convection cannot be excluded under ambient conditions, we find that directing thermal radiation accelerates the cooling process and renders fast cooling below room temperature possible. By introducing an elliptical mirror, we enhance the factor by almost 2 orders of magnitude. Also, thermal gradients are introduced on a sample, which are the direct result of the temperature landscape and the optical system. The observed temperature gradient of ~ 0.2 °C/mm, however, is highly dependent on the thermal properties of the sample and can be enhanced and shaped by different geometries or by choosing a less thermally conductive sample, which we are going to investigate in future work. Since our approach is contactless, we can achieve sample cooling with minimal disturbance, which we have demonstrated in a proof-of-concept experiment by realizing supercooling of liquid hexadecane in a microfluidic chip.

Spatially structured radiative cooling yields high potential for various applications in science and technology, where a contactless and therefore non-invasive temperature manipulation is desired. Such applications may include the control of chemical reaction kinetics and crystallization processes or biological studies in which thermal gradients in tissue or cell culture must be introduced while maintaining sterile conditions. Enzyme activity, gene expression levels and reaction–diffusion systems in cells, for example, are highly temperature dependent^54^. Localized heating has been applied in numerous studies in these research areas^26^, but localized cooling has been widely neglected. Furthermore, the optical scanning of cooling traces may be possible by adapting the optical system. By creating more complex temperature landscapes, various cooling patterns can be introduced. In the future, we will further investigate the experimental possibilities that are offered by the directed radiative cooling approach for physical, chemical and life-science applications.

## Materials and methods

A metal oxide coated aluminum film (“Metal Velvet”,* Acktar, Kiryad-Gat, Israel*; emissivity ε = 0.985 for λ = 1–10 µm) was mounted inside a cryostat (*Oxford Instruments, Barrington, New Jersey, USA*) and cooled down with liquid nitrogen. This sample served as the cold central region. The cryostat was sealed with a germanium window (*Thorlabs, Newton, New Jersey, USA*) that is transparent for light in a wavelength range between λ = 7–12 µm (Supporting Information, Figure S1) and therefore suitable for transmitting thermal radiation. For the first experiment, the room temperature emitter sample was either an identical metal oxide coated aluminum film or a borosilicate substrate that was coated with black paint.

An elliptical mirror (*E120-100, Optiforms, Temecula, California, USA*; diameter of 7.07 cm) with a bare gold coating (reflectance ≥ 0.985 for λ = 9–12 µm) was used to focus and reflect the thermal radiation between the room temperature sample and the cold central region of the temperature landscape. Temperature measurements and thermal imaging were performed using a long-wave infrared thermal camera (*FLIR, Wilsonville, Oregon, USA; A655sc*) with a temperature resolution < 30 mK. Acrylic glass sticks with low thermal conductivity were glued to the back of the sample and used as handles for positioning the substrate in the focus of the elliptical mirror.

Radiative cooling of hexadecane (C_16_H_34_) (*Sigma Aldrich, St. Louis, Missouri, USA*) was performed in a home-built microfluidic chip where acrylic glass is sandwiched in between two 170 µm thick glass substrates. Five independent reservoirs were drilled into the acrylic glass in a cross pattern with one reservoir in the center and four at the edges. To conduct the experiment, each reservoir was filled with 30 µL hexadecane and the sample was sealed from both sides. The glass substrate in the back of the chip is painted black for higher contrast.

## Supplementary Information

Supplementary Information.

## Data Availability

The datasets generated during and/or analyzed during the current study are available from the corresponding author on reasonable request.
