# Supplementary material for: Contactless and spatially structured cooling by directing thermal radiation
Source: Sci Rep. 2021 Aug 10;11:16209. doi: 10.1038/s41598-021-95606-2 (PMC8355347; doi:10.1038/s41598-021-95606-2)
Supplement: Supplementary file 1 — Supplementary Information. [file 41598_2021_95606_MOESM1_ESM.pdf]

Supporting Information

-

Contactless and spatially structured cooling by directing thermal radiation

Nicola M. Kerschbaumer[1], Stefan Niedermaier[1] Theobald Lohmüller[1] and Jochen Feldmann[1]

[1]Chair for Photonics and Optoelectronics, Nano-Institute Munich, Department of Physics, Ludwig-Maximilians-Universität (LMU), Königinstraße 10, 80539 Munich, Germany

1. Transmission spectrum of the germanium window.

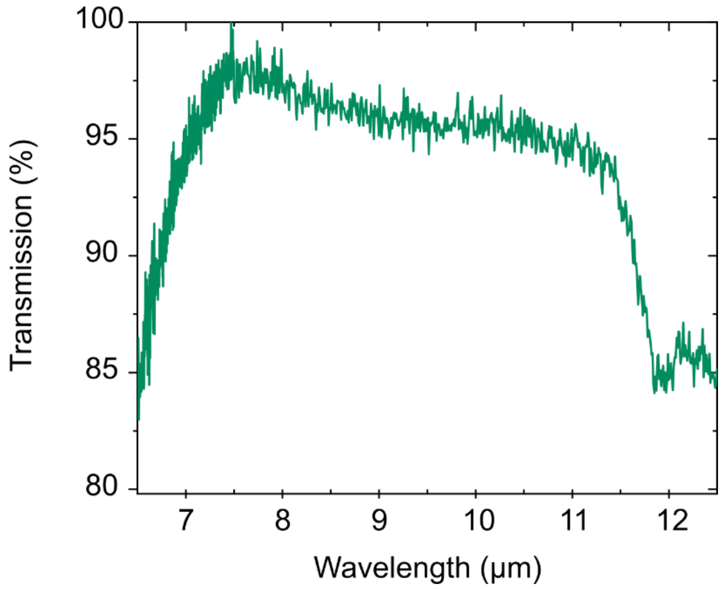

Figure S1: Transmission of the germanium window placed in front of the cryostat. The data is kindly provided by Thorlabs GmbH.

2. Mathematical analysis of the change of the view factor

With the values of the mirror dimensions as provided by the manufacturer, the following calculations have been performed to estimate the improvement of the view factor (F_V) by the addition of an elliptical mirror. The calculated values for each situation (with and without the mirror) are summarized in Table S1. From the focus point F_1 on the sample, the entire surface area A_{total} of a half-sphere with radius 10.16 cm (focal length between F_1 and F_2) is 648.59 cm^2.

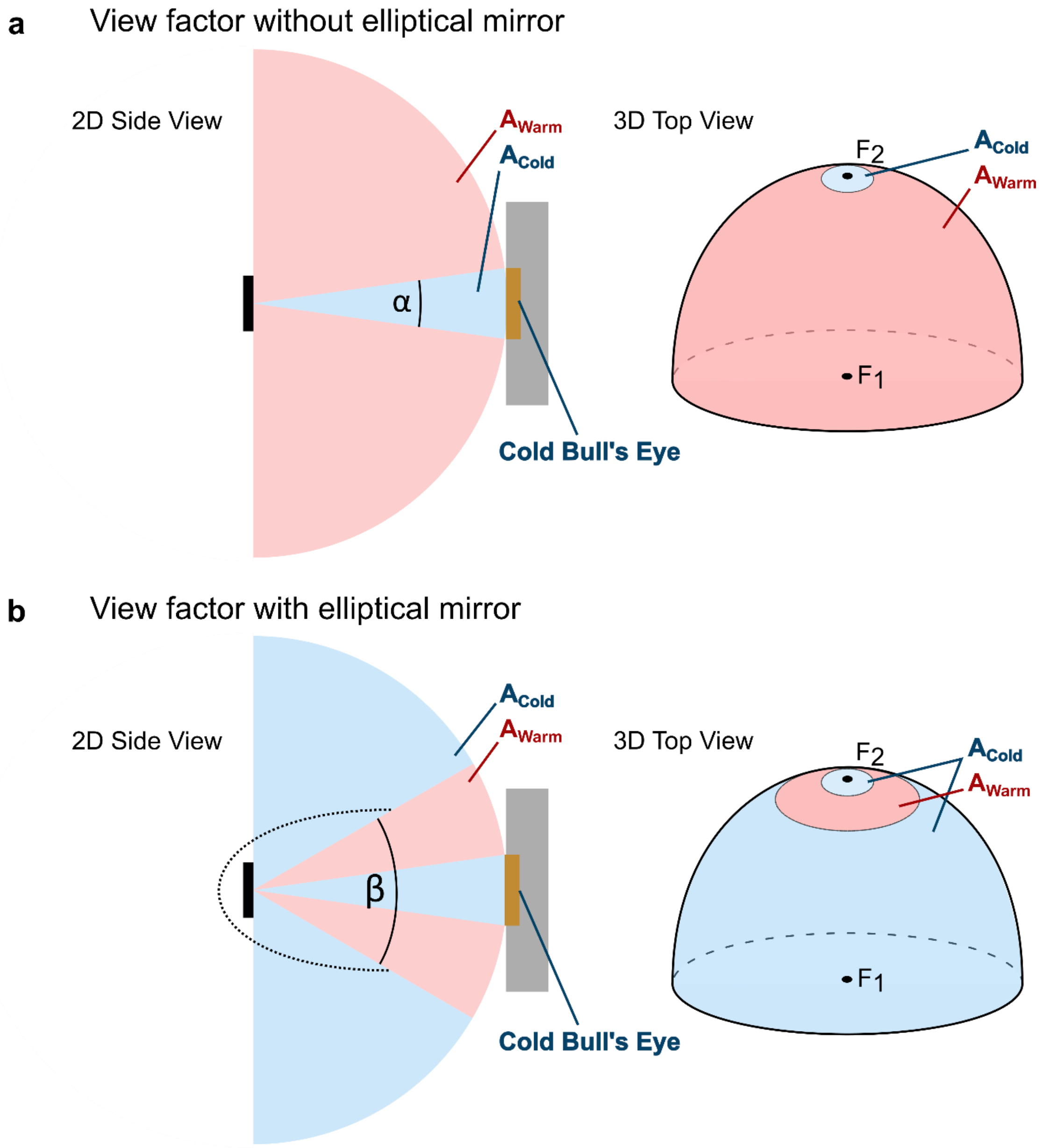

Figure S2: Depiction of the view factor without **a)** and with **b)** the elliptical mirror. The sample emits thermal radiation equally in all directions. We are considering the view factor F_V for the focal point F_1 on the sample. The light red describes the section of the view factor where radiation leaving the warm sample at F_1 does not strike the cold bull's eye on the cryostat window. **a)** Without the elliptical mirror, only the radiation emitted within the opening angle α is reaching the cold sample. The spherical cap of the half sphere corresponding to the opening angle is shown on the right, illustrating the surface areas of warm (A_{warm}) and cold (A_{cold}) that determine the view factor from point F_1. **b)** Introducing an elliptical mirror improves the view factor significantly, since a larger proportion of the radiation emitted at a solid angle is reflected to the cold bull's eye. In this configuration, only the thermal radiation emitted with the angle β-α does not contribute to the view factor.

View factor without the elliptical mirror:

When placing the sample at the focal point of the elliptical mirror, the only "cold region" that the sample is exposed to is the surface area of the germanium window of the cryostat. In this case, there is no structured cooling taking place as the sample cools down homogeneously. Considering the dimensions of the window, only 0.78% of thermal radiation emitted at the focus F_1 strikes the cold substrate (Table S1).

View factor with the elliptical mirror:

With an elliptical mirror, the proportions of the warm and cold radiation that determine the view factor change significantly. By considering the focal length and mirror dimensions, only a small fraction of thermal radiation that is emitted at an angle β-α is not directed by the mirror. This results in a large increase of F_v to 72.14%.

Value	Without mirror	With mirror
**A_{cold} [cm^2]**	5.07	467.88
**A_{warm} [cm^2]**	643.52	180.71
**$F_v = A_c/(A_{c+}A_w)$**	0.78%	72.14%

Table S1: Calculated values for the surface areas of the cold and warm sections of the view factor emphasize significance of the elliptical mirror.

3. Analysis of temperature decay curves.

For the measurement of the hexadecane in the microfluidic chamber, the temperature decay was fitted bi-exponentially. The fit equation is given by:

$$T(t) = a_1 * e^{-\kappa_1 t} + a_2 * e^{-\kappa_2 t} + c$$

Fit parameters:

Value	Position 1	Position 2
**a_1**	2.97	4.21
**a_2**	10.5	8.57
**κ_1**	0.138	0.0241
**κ_2**	0.00195	0.00190
**c**	11.0	11.8
**R^2**	0.999	0.999

Table S2: Fit parameters of the bi-exponential fit for supercooling of hexadecane.

The bi-exponential fit describes both, the exponential Newton's law of cooling due to convection and conduction, as well as the radiative heat transfer part. The weights a_1 and a_2 for both fits are of similar values and allowing us to compare the fits. Looking at the cooling rates κ_1 and κ_2, it is clearly evident that the fast rate κ_1 is approximately 5 times faster for position 1 than it is at position 2. The slower

rate κ₂ and the added constant c is almost identical for both curves. High R^2 values for both curves validate the fit.

As shown in Fig. S3, the data is fitted until the crystallization of hexadecane takes place, which is indicated by the vertical temperature jump. The temperature will further decrease in both positions 1 and 2. By considering a mono-exponential fit for the later time points, the offset values to which the exponents converge to have been extracted. For position 1 this value amounts to 11.08 °C, whereas a value of 11.94 °C is obtained for position 2. Position 1 is slightly colder, as it is located at the center of the focus of the elliptical mirror. At this position, the influence of the radiative cooling is strongest.

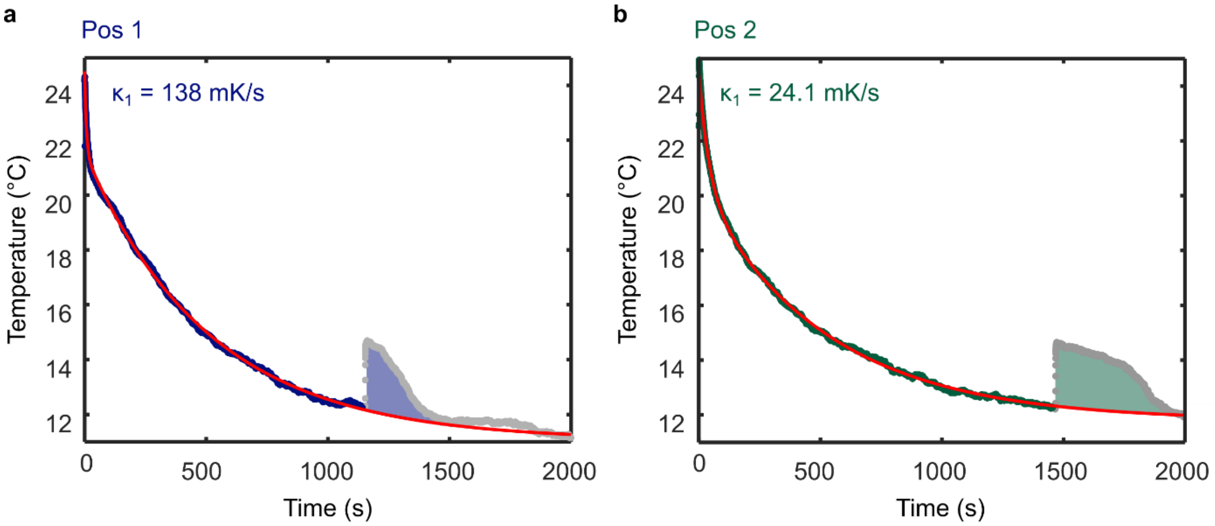

Figure S3: Bi-exponential fits applied to the temperature curves measured at position (Pos) 1 (**a**) and 2 (**b**). Only the blue (Pos 1) or green (Pos 2) points of temperature measurements are fitted (fit shown in red). The greyed-out data points are not considered for the fit. The colored-in areas show the region considered for the calculation of the half-life times.

Determination of the half-life time:

The half-lives were calculated by considering the time it takes for the sample after spontaneous crystallization to cool down back to half the value of the temperature before crystallization.
